# Gut Microbiome Composition Remains Stable in Individuals with Diabetes-Related Early to Late Stage Chronic Kidney Disease

**DOI:** 10.3390/biomedicines9010019

**Published:** 2020-12-29

**Authors:** Ashani Lecamwasam, Tiffanie M. Nelson, Leni Rivera, Elif I. Ekinci, Richard Saffery, Karen M. Dwyer

**Affiliations:** 1 Epigenetics Research, Murdoch Children’s Research Institute, VIC 3052, Australia; richard.saffery@mcri.edu.au; 2 Department of Endocrinology, Austin Health, VIC 3079, Australia; elif.ekinci@unimelb.edu.au; 3 School of Medicine, Faculty of Health, Deakin University, VIC 3220, Australia; leni.rivera@deakin.edu.au (L.R.); karen.dwyer@deakin.edu.au (K.M.D.); 4 Menzies Health Institute Queensland, Griffith University, QLD 4222, Australia; tiffanie.nelson@gmail.com; 5 Department of Medicine, University of Melbourne, VIC 3010, Australia; 6 Department of Paediatrics, University of Melbourne, VIC 3010, Australia

**Keywords:** chronic kidney disease, diabetes, dysbiosis, gut microbiome, microbiota

## Abstract

(1) Background: Individuals with diabetes and chronic kidney disease display gut dysbiosis when compared to healthy controls. However, it is unknown whether there is a change in dysbiosis across the stages of diabetic chronic kidney disease. We investigated a cross-sectional study of patients with early and late diabetes associated chronic kidney disease to identify possible microbial differences between these two groups and across each of the stages of diabetic chronic kidney disease. (2) Methods: This cross-sectional study recruited 95 adults. DNA extracted from collected stool samples were used for 16S rRNA sequencing to identify the bacterial community in the gut. (3) Results: The phylum Firmicutes was the most abundant and its mean relative abundance was similar in the early and late chronic kidney disease group, 45.99 ± 0.58% and 49.39 ± 0.55%, respectively. The mean relative abundance for family Bacteroidaceae, was also similar in the early and late group, 29.15 ± 2.02% and 29.16 ± 1.70%, respectively. The lower abundance of Prevotellaceae remained similar across both the early 3.87 ± 1.66% and late 3.36 ± 0.98% diabetic chronic kidney disease groups. (4) Conclusions: The data arising from our cohort of individuals with diabetes associated chronic kidney disease show a predominance of phyla Firmicutes and Bacteroidetes. The families Ruminococcaceae and Bacteroidaceae represent the highest abundance, while the beneficial Prevotellaceae family were reduced in abundance. The most interesting observation is that the relative abundance of these gut microbes does not change across the early and late stages of diabetic chronic kidney disease, suggesting that this is an early event in the development of diabetes associated chronic kidney disease. We hypothesise that the dysbiotic microbiome acquired during the early stages of diabetic chronic kidney disease remains relatively stable and is only one of many risk factors that influence progressive kidney dysfunction.

## 1. Introduction

The human gut harbors a complex community in excess of 100 trillion microbial cells that constitute the gut microbiota. They form a dynamic and symbiotic ecosystem that is in constant interaction with the host metabolism influencing nutrition, physiology, and immune function [1,2]. The microbial diversity increases and converges toward an adult-like microbiota by the end of the first 3–5 years of life [3]. Thus once established, the composition, function, and structure of the intestinal microbiota is relatively stable throughout the life course in healthy individuals [4]. This is despite being adaptive to the biochemical milieu of the gastrointestinal (GI) tract and changes in nutrient availability, which represent pivotal regulators of bacterial metabolism [4]. The bacterial phyla Firmicutes and Bacteroidetes constitute the majority of a healthy intestinal microbiome [5]. The composition of this bacterial diversity, however, appears to change in non-communicable diseases such as obesity [6], as well as for those with type 2 diabetes [7].

Diabetes and associated micro and macrovascular complications have reached pandemic proportions, with a global prevalence of 463 million [8]. More than 40% of people with diabetes develop diabetes-associated chronic kidney disease (CKD), which remains the leading cause of end stage kidney disease (ESKD) world-wide, requiring long-term dialysis [9]. Cardiovascular disease is the leading cause of death in individuals with CKD, in part due to persistent low-grade inflammation [10,11]. It is unsurprising then that diabetic CKD is now considered to be a global health issue.

Several studies have reported compositional change in intestinal microbiota in humans with diabetes compared to control subjects [12], and examples include reduced proportions of phylum Firmicutes, class Clostridia in those with type 2 diabetes compared to the healthy controls [12]. It has also been shown that individuals with diabetes generally exhibit reduced bacterial diversity characterised by a reduction of butyrate-producing bacteria such as Faecalibacterium prausnitzii and Roseburia intestinalis together with an increase in opportunistic pathogens [13].

Research in experimental animal models of chronic kidney disease (CKD) [14,15] and in patients with varying stages of CKD [16,17] demonstrate alterations to the normal gut microbiome. Chronic disease states such as diabetic CKD, via multiple pathophysiologic mechanisms, cause a state of “intestinal dysbiosis” of gut microbiota that may result in a systemic inflammatory response [18]. The hallmark changes of intestinal dysbiosis are a reduction of beneficial saccharolytic microbes producing short-chain fatty acids (SCFA) and in the case of CKD, an increase in proteolytic bacteria such as *Clostridium* spp. and *Bacteroides* spp. [18]. It is known that people with kidney disease have a higher level of serum urea or blood urea nitrogen (BUN), due to impaired kidney clearance of these uremic toxins and increased intestinal urea content. This increase has been proposed to lead to high ammonia production from bacteria-mediated urea hydrolysis, which in turn raises luminal pH and enhances the overgrowth of proteolytic species such as *Clostridium* species in CKD [19]. This ‘uremic milieu’ is not present in individuals with only diabetes (non-CKD) and therefore these individuals do not demonstrate an increase in Clostridium species. Given that more than 90% of the entire population of a healthy human gut microbiota are represented by two predominant phyla, namely Firmicutes and Bacteroidetes, when individuals with diabetes are compared with their healthy controls, there is a compositional change of the microbiome which consists of reduced Firmicutes in those with diabetes compared to healthy subjects.

A further hallmark of intestinal dysbiosis is the reduction of beneficial saccharolytic microbial families, such as Prevetoellaceae, that produce short-chain fatty acids such as butyrate. These short-chain fatty acids help maintain the integrity of the intestinal barrier, and are reduced in individuals with CKD compared to healthy subjects [20]. Individuals with diabetes are often clinically associated with the metabolic syndrome. In a human study evaluating the diversity of intestinal microbiota with aspects of the metabolic syndrome, the authors demonstrated that poor microbiota diversity was associated with obesity, insulin resistance, hepatic steatosis, and low-grade inflammation [21]. They showed that such subjects had a more pro-inflammatory microbial profile, characterised by a reduction of butyrate-producing bacteria [21].

Despite the disease burden, the pathophysiologic mechanisms underpinning the progression of diabetic CKD are poorly understood. Diabetic CKD is characterised by hypertension, albuminuria, and progressive decline in kidney function, measured as a change in the estimated glomerular filtration rate (eGFR). The role of the gut microbiome in the progression of diabetic CKD has emerged as an area of scientific interest [22]. Individuals with diabetes and chronic kidney disease display gut dysbiosis when compared to controls [16,23]. However, it is unknown whether there is a change in this dysbiosis across the stages of diabetic CKD; therefore, the aim of this study was to undertake a cross-sectional analysis of gut microbiome profiles of patients with early and late diabetes associated CKD to identify possible microbial differences between these two groups and across each of the stages (1–5) of diabetic CKD.

## 2. Materials and Methods

### 2.1. Participants

This prospective, cross-sectional study recruited 95 adults, over a 6 month period, at the time of their outpatient endocrinology clinic visit. The patients consented to providing a stool sample for DNA isolation, in addition to their routine blood tests at the outpatient visit. These patients had diabetes with varying stages (stages 1–5) of chronic kidney disease. Patients were divided into 2 distinct groups: ‘early CKD’ and ‘late CKD’. The early diabetic CKD group was defined as participants with diabetes who had stage 1, 2, or 3a CKD, while the late diabetic CKD group was defined as participants with diabetes who had stage 3b, 4, or 5 CKD. Diabetes-associated chronic kidney disease is the preferred terminology encompassing the range of histological kidney lesions that may be observed [24]. CKD is defined by a gradual decline in kidney function, measured as a change in the estimated glomerular filtration rate (eGFR), with or without albuminuria. Where available, albuminuria was evaluated by measuring the urinary albumin-to-creatinine ratio (ACR) in a spot urine sample at the same time of provision of their venous blood sample. Microalbuminuria was defined as an ACR of 30–300 mg/g and macroalbuminuria defined as an ACR > 300 mg/g [25]. Six stages of CKD are recognized—Stage 1 (eGFR ≥ 90 mL/min/1.73 m^2^), 2 (eGFR 60–89 mL/min/1.73 m^2^), 3a (eGFR 45–59 mL/min/1.73 m^2^), 3b (eGFR 30–44 mL/min/1.73 m^2^), 4 (eGFR 20–29 mL/min/1.73 m^2^), and 5 (eGFR < 20 mL/min/1.73 m^2^). The risk of death from any cause, cardiovascular events, or hospitalization increases exponentially from Stage 3b onwards [26].

Data collection occurred only at one time point and included information on blood pressure, medical comorbidities, duration of diabetes, the stage of CKD, and its associated complications, medications, and pathology results. Anthropometric data and stool samples were collected on the day of the clinic visit while the remainder of the patient’s information was gathered via access to the Austin Health’s electronic medical record. All of this selected information was then entered into a database specific for this research study for subsequent analysis. There were 70 participants in the early diabetic CKD group and 25 in the late group. Participants provided written informed consent and the study was approved by the Human Research Ethics Committee of Austin Health, Victoria, Australia (HREC/17/Austin/166, project number ND 17/166, with HREC approval on 13/07/2017) and the Human Research Ethics Committee of Deakin University, Australia. The procedures followed were in accordance with the Helsinki Declaration of 1975, as revised in 2013.

### 2.2. Stool Collection

Following collection by the donor in a 50 mL specimen container, samples were aliquoted approximately 0.5–1 g into smaller 1.5 mL Eppendorf tubes before freezing at −80 °C for future DNA extraction.

### 2.3. DNA Extraction

DNA was extracted using the Qiagen QIAamp^®^ DNA Stool Mini Kit (Ref 51504, Hilden, Germany) according to the manufacturer’s protocol. DNA quantification and purification was assessed using a Qubit fluorometer (Invitrogen). DNA extractions from the stool aliquots were performed at the completion of patient recruitment.

### 2.4. DNA Microbiome Profiling and Quality Control

PCR amplification and sequencing was performed by the Australian Genome Research Facility (AGRF, Melbourne, Australia). PCR amplicons were generated using the primers and conditions outlined in Table 1. Thermocycling was completed with an Applied Biosystem 384 Veriti and using AmpliTaq Gold 360 mastermix (Life Technologies, Mulgrave, VIC, Australia) for the primary PCR. The first stage PCR was cleaned using magnetic beads, and samples were visualised on 2% SYBR Agarose E-Gel (Thermo-Fisher, Mulgrave, VIC, Australia). A secondary PCR to index the amplicons was performed with TaKaRa Taq DNA Polymerase (Takara Bio USA, Inc., Mountain View, CA, USA). The resulting amplicons were cleaned again using magnetic beads and were quantified with a QuantiFluor fluorometer (Promega, Madison, WI, USA) prior to normalisation. Samples were combined in equimolar concentrations prior to cleaning a final time using magnetic beads to concentrate the pool and then measured using a High-Sensitivity D1000 Tape on an Agilent 2200 TapeStation System. The pool was diluted to 5 nM and molarity was confirmed again using a High-Sensitivity D1000 Tape. This was followed by sequencing on an Illumina MiSeq (San Diego, CA, USA) with a V 3, 600 cycle kit (2 × 300 base pairs paired-end).

Paired-ends reads were assembled by aligning the forward and reverse reads using PEAR (version 0.9.5) [27]. Primers were identified and trimmed. Trimmed sequences were processed using Quantitative Insights Into Microbial Ecology (QIIME 1.8) [28]. USEARCH (version 7.1.1090) [29,30] and UPARSE [31] software. Using USEARCH, sequences were quality filtered, then full length duplicate sequences were removed and were sorted by abundance. Singletons or unique reads in the data set were discarded. Sequences were clustered followed by chimera filtering using the “rdp_gold” database as the reference. To obtain the number of reads in each operational taxonomic unit (OTU), reads were mapped back to OTUs with a minimum identity of 97%. Using QIIME, taxonomy was assigned using Greengenes database (version 13_8, August 2013) [32].

### 2.5. Data Cleaning, Normalisation and Statistical Analysis

The categorical variables for the patient characteristics were tested using Fisher’s Exact and the continuous variables were tested with Wilcoxon-signed rank test.

Data were pruned to remove representatives classified to Archaea (N = 4), Chloroplast (N = 42), and 22 unassigned OTUs implemented in the package ‘phyloseq’ in the R statistical program version 1.26.1 [33]. We also removed the OTUs with a prevalence of less than two, which made the logged counts per sample more evenly distributed. The remaining 1818 taxa were classified to the Kingdom Bacteria with 69.03% assigned to the phylum level. We visualised relative abundance of bacteria (abundance > 2%) in different sample types, classified to the phylum and genus level.

We calculated the number of observed OTUs and compared the alpha diversity (within the sample) of individuals grouped by the CKD stage using the Shannon Index [34], which accounts for both abundance and evenness of the taxa present. Boxplots of alpha diversity indices were generated using the *boxplot* command in base R. Because our data have potential outliers and were not normally distributed, differences between sample group means were tested with the non-parametric Kruskal-Wallis test, using the command *kruskal.test* [35].

For beta diversity (between samples), we used a log transformation of data scaled to read depth per sample and then calculated and plotted weighted UniFrac [36], which provides a measure of relative abundance and phylogenetic dissimilarity. A smaller UniFrac distance between two samples indicates a higher similarity among the two microbial communities [37]. UniFrac distances were visualised using Principle Coordinate Analysis (PCoA) plots using commands from the package ‘phyloseq’ [33]. Principal coordinate analysis (PCoA) is a dimensionality reduction method that illustrates the relationship between samples depending on the distance matrix and visualises the unsupervised grouping pattern of a complex data set, such as the microbiome. We used the *adonis* command from the package ‘vegan’ [38] to perform permutational multivariate analysis of variance (PERMANOVA) to check whether the microbial communities of each sample groups were significantly different [34]. Results for all statistical tests were considered to be significant where *p*-values <0.05.

## 3. Results

Clinical and Biochemical Characteristics

**Figure 1:**
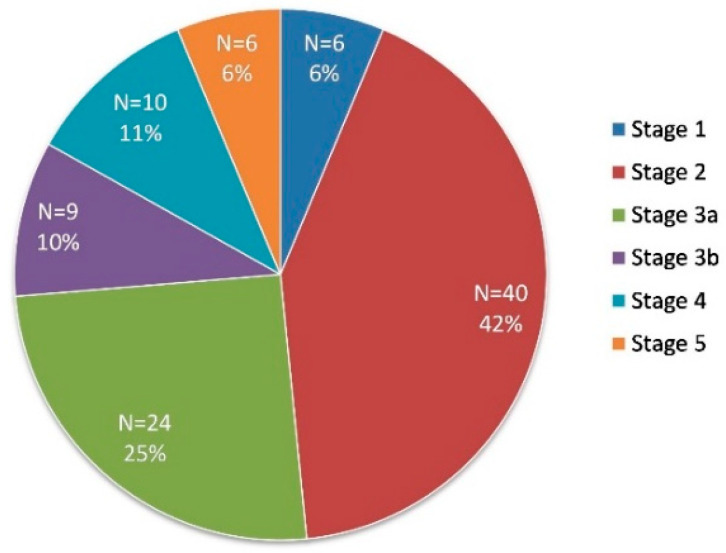
Proportion of patients in each stage of diabetic chronic kidney disease (CKD).

After sample quality control, data were available for 95 patient samples. The clinical and biochemical characteristics of the 95 study population are shown in Table 2. The mean eGFR was 67.51 mL/min/1.73 m^2^ in the early diabetic chronic kidney disease group (consisting of stage 1, 2, and 3a CKD), and 24.48 mL/min/1.73m^2^ in the late diabetic chronic kidney disease individuals (consisting of stage 3b, 4, and 5). This reached statistical significance with a *p* value < 0.001 (Wilcoxon Signed Rank Test). The mean age in the early CKD group was significantly younger at 66.24 years compared with 72.68 years in the late CKD group (*p* value = 0.01). The proportion of the 95 recruited patients in each stage of diabetic CKD is illustrated in Figure 1.

**Figure 2:**
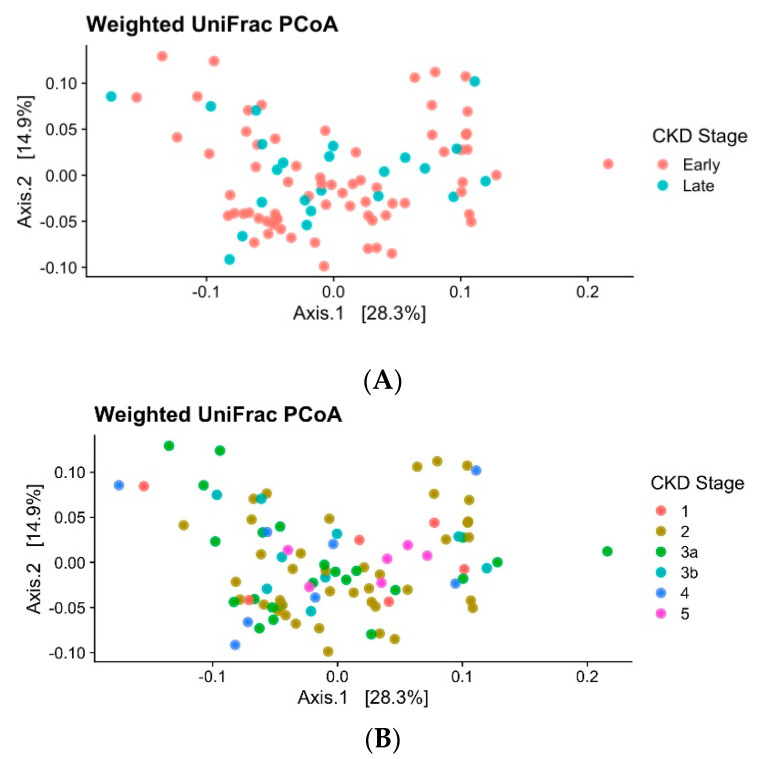
(**A**) Principal coordinate analysis (PCoA): Ordination plots display the dissimilarity of the gut bacterial community from individuals with CKD. Plots are labelled to show CKD grouped into classifications of early (1 to 3a) and late (3b to 5) stages. Data were based on weighted UniFrac of log-transformed relative abundances. (**B)** Ordination plots display the dissimilarity of the gut bacterial community from individuals with CKD. Plots are labelled to show CKD at fine-scale groups. Data were based on weighted UniFrac of log-transformed relative abundances.

The beta diversity indices are used to describe ecological diversity between microbial community samples. The beta diversity of the gut microbiome did not significantly differ between early versus late groups of diabetic CKD individuals or between each of the stages of diabetic CKD when compared with another (PERMANOVA, *p* value = 0.70, Figure 2A,B).

**Figure 3:**
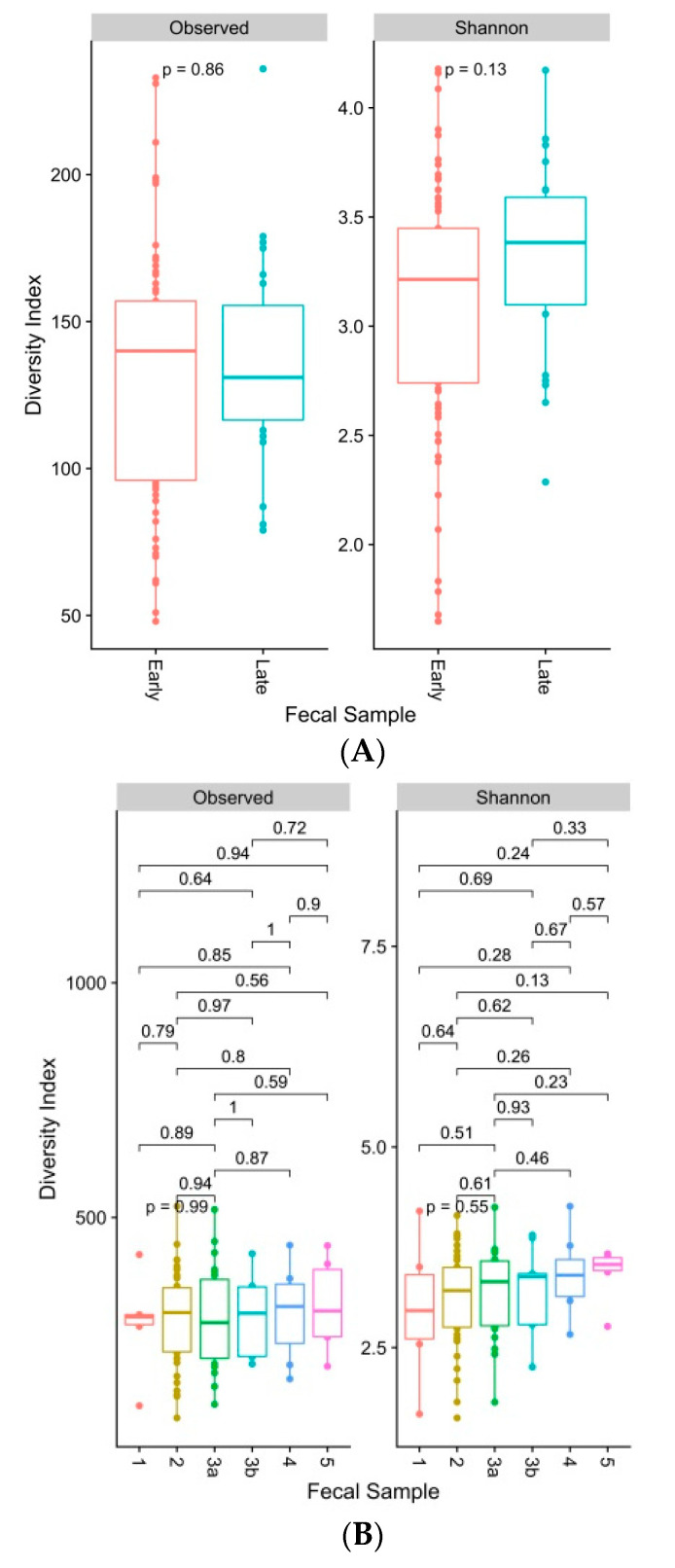
(**A**)**.** Box and whiskers plots display number of observed bacterial operational taxonomic units (OTUs) and Shannon’s diversity index of faecal samples grouped by individuals with early (1 to 3a) and late (3b to 5) stages of CKD. Boxes represent the interquartile range (IQR) between the first and third quartiles (25th and 75th percentiles, respectively) and the horizontal line inside the box defines the median. Whiskers represent the lowest and highest values within 1.5 times the IQR from the first and third quartiles, respectively. Solid dots (●) outside the whiskers indicate greater than 1.5 times and less than 3 times the IQR. Indices were generated from raw, untrimmed, rarefied data. Significance testing between groups were conducted using a Kruskal-Wallis Test. Results were considered significant where *p* ≤ 0.05. (**B).** Box and whiskers plots display number of observed bacterial operational taxonomic units (OTUs) and Shannon’s diversity index of faecal samples grouped by individuals at CKD stages 1, 2, 3a, 3b, 4, and 5. Boxes represent the interquartile range (IQR) between the first and third quartiles (25th and 75th percentiles, respectively) and the horizontal line inside the box defines the median. Whiskers represent the lowest and highest values within 1.5 times the IQR from the first and third quartiles, respectively. Solid dots (●) outside the whiskers indicate greater than 1.5 times and less than 3 times the IQR. Indices were generated from raw, untrimmed, rarefied data. Significance testing between groups were conducted using a Kruskal-Wallis Test. Results were considered to be significant where *p* ≤ 0.05.

The alpha diversity indices are commonly used to describe ecological diversity within the microbial community samples. The Shannon index considers both the species richness and evenness. The alpha diversity of the gut microbiome did not differ significantly between early versus late groups of diabetic CKD individuals (Shannon index, Kruskal-Wallis Test, *p* value ≤ 0.05) or between each of the stages of diabetic CKD when compared with another (Figure 3A,B).

**Figure 4:**
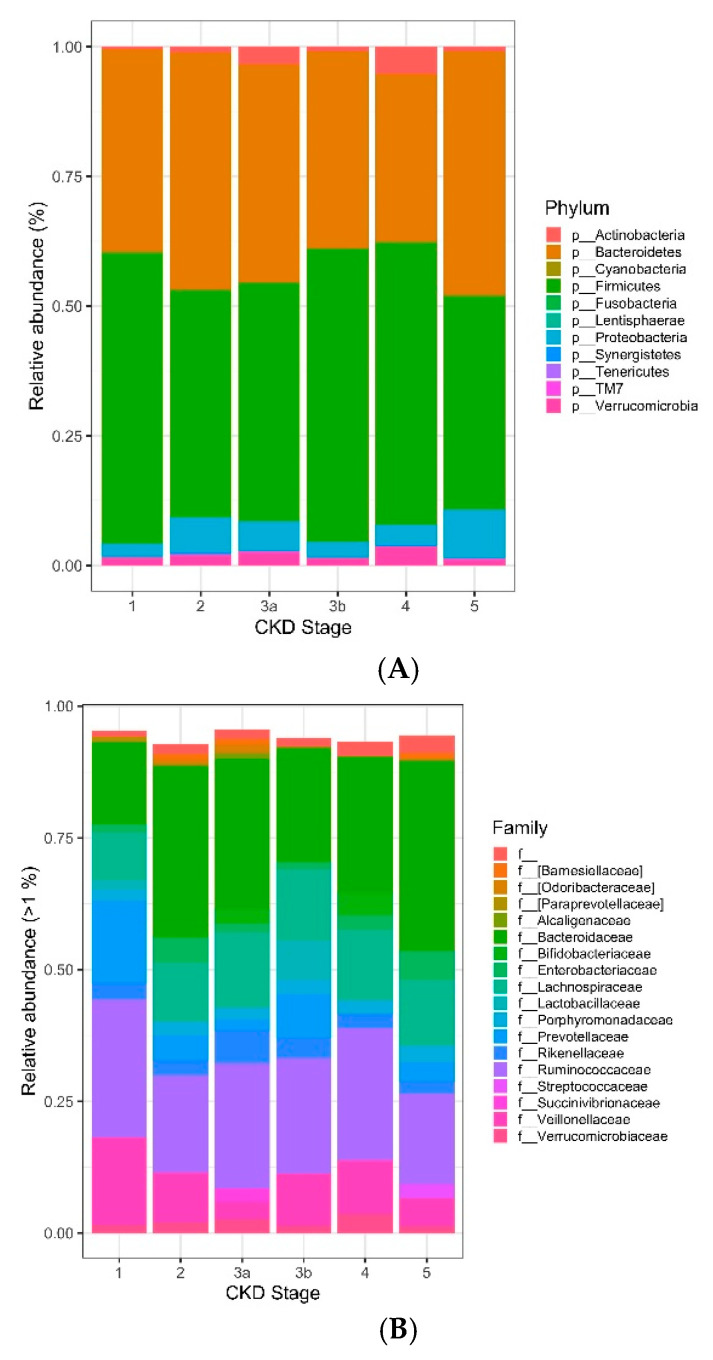
(**A**)**.** Stacked bar charts display the average relative abundance of bacterial operational taxonomic units (OTUs) identified to the taxonomic level of phylum in the gut microbiome of individuals with different stages of CKD disease. Abundances were calculated from trimmed data to remove OTUs that were prevalent in <2 samples with <2 counts in the complete dataset. (**B**)**.** Stacked bar charts display the average relative abundance of bacterial operational taxonomic units (OTUs) identified to the taxonomic level of family in the gut microbiome of individuals with different stages of CKD disease. Abundances were calculated from trimmed data to remove OTUs that were prevalent in <2 samples with <2 counts in the complete dataset.

The majority (>85% average relative abundance) of bacterial operational taxonomic units (OTUs) identified to the taxonomic level of phylum in the gut microbiome of individuals with different stages of CKD disease were represented by the phyla Bacteroidetes and Firmicutes. The phylum Firmicutes was the most abundant and its mean relative abundance was similar in the early (stage 1, 2, and 3a) and late (stage 3b, 4, and 5) CKD group, accounting for 45.99 ± 0.58% in early CKD and 49.39 ± 0.55% in late CKD. Likewise, the mean relative abundance for phylum Bacteroidetes was similar in early diabetic CKD accounting for 42.86 ± 1.40% and 41.20 ± 1.12% in the late diabetic CKD group (Figure 4A). At the family level, Bacteroidaceae, and Ruminococcaceae represented the highest abundance of OTUs across the early and late CKD groups while Prevotellaceae had the lowest abundance across all stages of diabetic CKD. Specifically, the mean relative abundance for family Bacteroidaceae, was similar in early and late diabetic CKD, accounting for 29.15 ± 2.02% in the early CKD group and 29.16 ± 1.70% in the late CKD group. The mean relative abundance of Ruminococcaceae was also similar in early and late CKD, consisting of 20.49 ± 0.61% in early CKD and 20.22 ± 0.44% in late CKD. The lower abundance of Prevotellaceae also remained similar across both early and late CKD, accounting for only 3.87 ± 1.66% in early CKD and 3.36 ± 0.98% in late diabetic CKD (Figure 4B).

**Figure 5:**
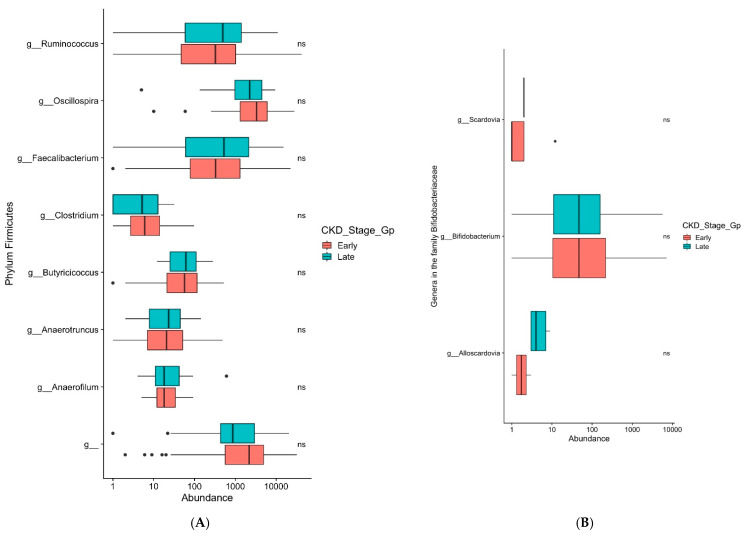

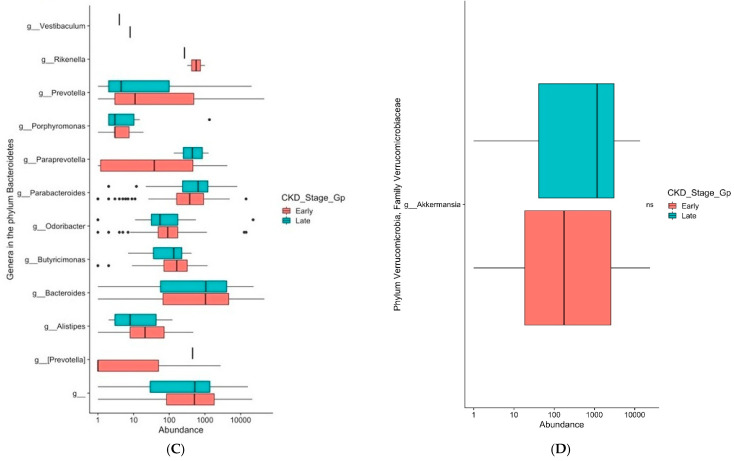
(**A**): Box and whiskers plots display a relative abundance of genera *Ruminococcus* and *Faecalibacterium* between early and late CKD groups. ns denotes non significance. (**B**): Box and whiskers plots display relative abundance of genus *Bifidobacterium* between early and late CKD groups. ns denotes non significance. (**C**): Box and whiskers plots display relative abundance of genus *Bacteroides* between early and late CKD groups. (**D**): Box and whiskers plots display relative abundance of genus *Akkermansia* between early and late CKD groups. ns denotes non significance.

**Figure 6:**
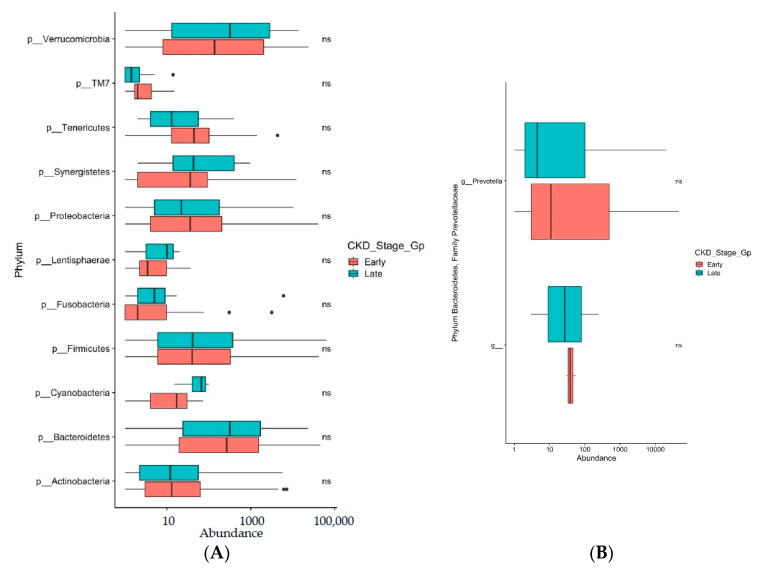
(**A**)**:** Box and whiskers plots display relative abundance of phyla Firmicutes and Actinobacteria between early and late CKD groups. ns denotes non significance. (**B**)**:** Box and whiskers plots display relative abundance of genus *Prevotella* between early and late CKD groups. ns denotes non significance.

The genera *Faecalibacterium, Bifidobacterium, Bacteroides,* and *Akkermansia,* which are known to be negatively associated with type 2 diabetes [39], did not demonstrate a significant difference in their relative abundance in either early or late diabetic CKD in our study (Figure 5A–D). The phyla Actinobacteria and Firmicutes, which have been shown to have colonic overgrowth in CKD patients [23], again showed no significant difference in relative abundance in either early or late diabetic CKD (Figure 6A). The gut microbiome in uremic animal models was associated with reduced composition of Prevotellaceae [23]. In our study, the genus *Prevotella* in the family Prevotellaceae showed no significant difference in relative abundance in either early or late diabetic CKD (Figure 6B).

## 4. Discussion

A significant body of literature provides evidence for the role of gut microbiota in chronic metabolic disease processes including type 2 diabetes [40]. Among the commonly reported findings, the genera of *Bifidobacterium, Bacteroides, Faecalibacterium, Akkermansia,* and *Roseburia* were negatively associated with type 2 diabetes, while the genera of *Ruminococcus, Fusobacterium,* and *Blautia* were positively associated with type 2 diabetes [39]. The intestinal microbiota can be highly adaptable to changes in the biochemical milieu, while recent studies by Vaziri et al. [23] have shown increased counts of aerobic and anaerobic bacteria in the small bowel of CKD patients together with a colonic overgrowth of Proteobacteria, Actinobacteria, and Firmicutes.

Diabetic CKD is a multi-systemic disease process with complex pathophysiological processes. Indeed the cause-consequence relationship between such a dynamic pathological process and associated changes to intestinal microbiota is difficult to differentiate. Factors known to be responsible for the dysbiosis in CKD include increased intestinal barrier permeability secondary to an inflammatory and uraemic milieu associated with CKD and subsequent translocation of pathogenic bacteria and bacterial endotoxins from the gut lumen into the bloodstream due to this increased gut permeability [41]. A majority of individuals with diabetes-associated CKD are often on broad-spectrum antibiotics due to increased risk of infections that arise from this condition. The use of such antibiotics leads to an imbalance between Firmicutes and Bacteroidetes. The bacterial diversity decreases as does the abundance of these bacteria during such treatment periods [42]. Furthermore, an important regulator of bacterial metabolism and gut dysbiosis is represented by nutrient availability and composition, in particular the ratio between undigested carbohydrates and protein. There are currently a number of CKD-associated processes leading to such a gut dysbiosis. As an example, protein absorption in the small intestine is impaired in CKD [43] leading to an increased amount of dietary protein in the colon and consequently a reduction in the colonic carbohydrate-to-protein ratio. This change in substrate availability may favour a shift from a healthy saccharolytic (*Bifidobacterium* and *Lactobacillus* species) to a more pathogenic proteolytic fermentation pattern. Although the mechanisms for dysbiosis in diabetes are less clearly understood, it has been shown that individuals with diabetes generally exhibit reduced bacterial diversity characterised by a reduction of butyrate-producing bacteria such as Faecalibacterium prausnitzii and Roseburia intestinalis together with an increase in opportunistic pathogens [13].

The ability to isolate gut dysbiotic causality due to diabetes associated CKD alone, remains a clinical challenge due to the presence of multiple comorbidities such as hypertension, obesity and vascular disease. Furthermore, therapeutic strategies in diabetic CKD include medications and dietary restrictions which as described above, can independently affect the gut microbiome [44,45]. Despite these complexities, it is reasonable to hypothesise that kidney disease and the gut microbiota may influence each other [18].

In particular, the gut microbiome differences between healthy individuals and those individuals with diabetic CKD have been documented [16,23,46]. With these data in mind, we undertook a cross-sectional analysis of DNA profiles in 95 stool samples from an Australian population with predominantly type 2 diabetes with varying stages (1–5) of CKD with the aim of answering the question of whether the gut microbiome changes across these different stages of diabetic CKD. As such, the primary end point was to identify potential differential microbiome profiles between groups with early versus late CKD. The basis for the cut-off of CKD stages 1, 2, and 3a into the early group and CKD stages 3b, 4, and 5 into the late group is because the risk of progressive renal dysfunction and cardiovascular disease increases significantly from CKD stage 3b onwards and clinical interventions, which aim to slow down CKD progression, are of particular importance.

The data has shown that the gut microbiome of individuals with early and late diabetic CKD and across all stages 1–5 of diabetic CKD are similar. Consistent with the literature [47], we have shown that there is a predominance of phylum Firmicutes and Bacteroidetes in our cohort (Figure 4A). In our study, we have clearly shown that the negatively associated genera with type 2 diabetes, causing dysbiosis, particularly *Faecalibacterium, Bifidobacterium, Bacteroides,* and *Akkermansia* [39] are present across the stages of CKD in similar amounts of relative abundance (Figure 5A–D). The positively associated genus *Ruminococcus* in individuals with type 2 diabetes [39], also shows no significant difference in its relative abundance in early or late stage diabetic CKD (Figure 5A). The proteolytic bacteria phyla Actinobacteria and Firmicutes, which have been shown to result in colonic overgrowth [23], were present across stages of CKD in similar relative abundance (Figure 6A). Further, when we examined the ecological diversity within the microbial sample of each stage of diabetic CKD and compared it against each of the other diabetic CKD stages, we still did not observe a significant difference (Figure 3B). These findings corroborate the known evidence about dysbiosis in disease states but additionally provide novel evidence to show that the acquired gut dysbiosis in early stage diabetic CKD, remains stable and persists through to the later stages of disease progression. These data build on the existing literature where the gastrointestinal microbial composition was examined in two CKD groups. Kai-Yu Xu et al. [16] examined the gut microbiome in 15 patients in a high GFR subgroup, defined as GFR ≥ 7 mL/min/1.73 m^2^, and a low GFR subgroup, defined as GFR ≤ 7 mL/min/1.73 m^2^ [16]. Whilst consistent with our data, it is difficult to draw too many conclusions from their study given small numbers within the subgroups and the use of GFR cut offs that do not reflect the clinical spectrum of the different stages of chronic kidney disease, as both subgroups fall within stage 5 or end stage kidney disease.

Our data has shown that the average relative abundance of bacterial phyla and family across stages 1–5 of diabetic CKD were mostly similar (Figure 4A,B). This is in keeping with data arising from a study by Yacoub et al., which evaluated the gut microbiome in individuals with polycystic kidney disease with varying stages of CKD [48]. Yacoub et al. performed a study in a highly select group of individuals with only polycystic kidney disease and 3 groups of CKD, without the confounding comorbidities of diabetes and hypertension, to examine the effect of varying degrees of renal insufficiency on the human gut microbiome. Despite small numbers of six patients in each of the 3 CKD groups, they did not demonstrate a difference in the operational taxonomic units (OTUs) at the phyla level across their 3 groups of varying kidney dysfunction [48].

A distinct gut microbiome with reduced Prevotellaceae families has been associated with CKD [23]. Consistent with this literature, our results have further demonstrated low mean relative abundance of beneficial Prevotellaceae in both the early (3.87 ± 1.66%) and late (3.36 ± 0.98%) stages of diabetic CKD (Figure 6B). *Prevotella* strains are classically considered to be commensal bacteria due to their extensive presence in the healthy human body. *Prevotella* is a genus with high genetic diversity within and between species, which could explain its abundance in human healthy microbiota [49]. As described earlier, these commensal bacteria produce short chain fatty acids (SCFAs), especially butyric acid, which is vital for maintaining gut health. The roles of SCFAs include but are not limited to producing intestinal epithelial nutrition and energy components [50], maintaining intestinal barrier functions [51], and reducing the severity of inflammation [52]. Similar to our study, Vaziri et al. found reduced abundance of the Prevotellaceae family in uremic animals [23].

In our study, there was no demonstrable difference in the operational taxonomic units (OTUs), at any of the taxonomic levels across all stages of diabetic CKD suggesting that dysbiosis appears early in diabetic CKD and persists through to the late stages of the disease thus raising the question of what role gut dysbiosis plays in the progression of diabetic CKD. We postulate that dysbiosis is one of a number of factors that may influence progression of diabetic CKD. Comorbid conditions such as hypertension, diabetes, and vascular disease, together with proteinuria and genetics are some of the contributors to the pathogenesis of chronic kidney disease [53]. More recently, there has been increasing evidence that the gene-environment interaction in determining the complex phenotype, otherwise known as epigenetics, is also a contributor to the pathogenesis of chronic kidney disease [54]. We hypothesise that gut dysbiosis, similar to the above heterogeneous risk factors, may confer a potential susceptibility factor in progression of CKD.

It is known that in gut dysbiosis, pathogenic bacteria overgrow and secrete increased amounts of lipopolysaccharides, peptidoglycans, and bacterial DNA into the host circulatory system which are detrimental to intestinal permeability [55]. Consequently, this results in activation of the intestinal-mucosa immune system [56] and the inflammatory cascade with production of factors such as interleukin (IL)-6, interferon γ (IFN- γ), and the tumor necrosis factor (TNF α) [57]. Such persistent immune activation is now considered as a major risk factor for CKD progression and cardiovascular complications [58]. The effect of gut dysbiosis on the production of inflammatory factors in CKD was investigated by Li F et al. [47], and their results suggest that microbiota dysbiosis may promote chronic systemic inflammation in CKD [47].

Diabetes occurs very commonly and is globally present in pandemic proportions [8] while diabetic chronic kidney disease is a common sequelae [9]. However, only a relatively small proportion of these individuals with CKD progress to end stage kidney disease (ESKD) over their life course [59]. We hypothesise that each of the traditional risk factors may be implicated in CKD development, however, the progressive nature of this disease, which occurs in the minority of all people with the condition, may be the result of the complex interaction of all these susceptibility factors, including gut dysbiosis (Supplementary Materials Figure S1).

One of the limitations of this study is its small sample size, especially the presence of only 25 patients with the late stages (3b–5) diabetic CKD resulting in a potential loss of the ability to recognise microbial trends. One of the reasons for this is that the study subjects were selected from an outpatient endocrine clinic, where there is a lesser propensity to see more advanced renal dysfunction. We acknowledge that the inherent problem of a small sample size in the late CKD group together with our large number of covariates, would result in a limited statistical power to detect microbiome differences as well as imprecision of the effect estimate and an elevated false positive rate. Other limitations include the cross-sectional nature of this study cohort, which meant that patient samples were collected at only one time point and not studied longitudinally. Furthermore, our data set did not contain information on antibiotic usage, which is known to have an impact on gut microbiome composition. Another significant limitation is that the dietary habits of the participants were not recorded which will invariably affect the outcome of results. There was also no healthy group, however, as described previously, the microbiome between healthy and diseased group has been well characterised and was not the purpose of this study.

Future directions in this area of research should include a longitudinal study of a larger number of patients in each of the stages of diabetic CKD (1–5), with a focus on the predominant form of Type 2 diabetes with information inclusive of dietary intake and medications, especially pertaining to antibiotic use.

This study raises some unexpected yet interesting questions about the microbiome in individuals with diabetes and varying kidney dysfunction. We know from current literature that there is a significant difference in the gut microbiome between healthy people and those individuals with diabetic CKD. However, within the limitations of this study, we did not observe a significant change in the microbiome across any of the six stages of diabetic CKD. We propose that once patients achieve a state of dysbiosis in early diabetic CKD, this dysbiosis remains relatively the same and could be considered a susceptibility risk factor, which together with other risk factors (traditional and novel), influence the progression to late stage diabetic CKD.

## 5. Conclusions

In conclusion, the data arising from our cohort of individuals with diabetes associated with CKD show a predominance of phyla Firmicutes and Bacteroidetes. The families Ruminococcaceae and Bacteroidaceae represent the highest abundance, while the beneficial Prevotellaceae family was reduced in abundance. The most interesting observation is that the relative abundance of these gut microbes does not change across the stages (1–5) of diabetic CKD, suggesting that this is an early event in the development of diabetes associated CKD. The negatively associated genera *Bifidobacterium*, *Bacteroides*, *Faecalibacterium,* and *Akkermansia* in relation to type 2 diabetes showed a similar relative abundance between early and late CKD groups, as did the positively associated *Ruminococcus*. We hypothesise that the dysbiotic microbiome acquired during the early stages of diabetic CKD remains relatively static and is only one of many risk factors that influence progressive kidney dysfunction. These findings warrant a further examination in larger patient cohorts followed longitudinally over time with multiple sampling and clinical data inclusive of information on dietary intake and medications. Only then will it be possible to robustly test whether the dysbiotic microbiome composition remains the same or significantly changes with the progression of diabetic chronic kidney disease.

## Figures and Tables

**Table 1:** Generation of amplicons using primers and conditions outlined (Australian Genome Research Facility, AGRF, Melbourne, Australia).

Target	Cycle	Initial	Disassociate	Anneal	Extension	Finish
16S: V3–V4	29	95 °C for 7 min	94 °C for 30 s	50 °C for 60 s	72 °C for 60 s	72 °C for 7 min
**Target**	**341F-806R**					
Forward Primer(341F)	CCTAYGGGRBGCASCAG					
ReversePrimer(806R)	GGACTACNNGGGTATCTAAT					

**Table 2:** Patient Clinical and Biochemical Characteristics.

Patient Characteristics	Mean Early CKD (Group 1)	SD	Mean Late CKD (Group 2)	SD	*p*-Value (Wilcoxon Signed Rank Test)	*p*-Value (Welch Two Sample t-Test)
Age (years)	66.24	10.22	72.68	10.21	0.01	0.01
Male (%)	80.70		19.30		0.09	
Type of Diabetes	0.83	0.42	0.92	0.28	0.29	0.22
Type 1 (%)	86.67		13.33			
Type 2 (%)	70.89		29.11			
Latent autoimmune diabetes in adults (LADA) (%)	100.00		0.00			
Duration of Diabetes (years)	18.81	11.63	21.00	10.99	0.32	0.41
Diabetic Retinopathy (%)	0.39	0.49	0.40	0.50	0.90	0.90
Cardiovascular Disease (%)	57.89		42.11		0.09	
Stroke/Transient Ischaemic Attack (TIA%)	0.13	0.34	0.12	0.33	0.92	0.91
Anxiety	6.77	4.22	8.32	4.98	0.30	0.17
Depression	5.64	3.67	7.00	3.74	0.11	0.12
Smoking quantity pack_year	9.83	13.12	6.60	11.70	0.22	0.26
Smoking status (%)	0.51	0.61	0.32	0.56	0.14	0.15
Non-smoker (%)	67.86		32.14			
Ex-smoker (%)	82.35		17.65			
Current-smoker (%)	80.00		20.00			
Body Mass Index (BMI)(kg/m^2^)	29.64	7.46	30.12	5.23	0.40	0.73
Systolic blood pressure (SBP) (mmHg)	127.83	27.37	135.04	29.32	0.74	0.29
Diastolic blood pressure (DBP) (mmHg)	73.30	15.91	71.72	9.86	0.21	0.57
Haemoglobin (Hb) (g/L)	132.66	16.92	117.72	12.72	0.00	0.00
Estimated glomerular filteration rate (eGFR) (mL/min/1.73 m^2^)	67.51	13.68	24.48	10.68	0.00	0.00
Glycated haemoglobin (HbA1c) (%)	7.68	1.95	6.86	3.35	0.62	0.25
Urine Albumin/Creatinine ratio (ACR)	3.37	10.52	33.65	122.81	0.97	0.23
Urine Protein/Creatinine ratio (PCR)	0.02	0.03	0.06	0.16	0.99	0.15

## Supplementary Materials

The following are available online at https://www.mdpi.com/2227-9059/9/1/19/s1, Figure S1: Risk factors for diabetic CKD and its progression
