# Supplementary figures and images for: Gut Microbiome Composition Remains Stable in Individuals with Diabetes-Related Early to Late Stage Chronic Kidney Disease
Source: Biomedicines. 2020 Dec 29;9(1):19. doi: 10.3390/biomedicines9010019 (PMC7824346; doi:10.3390/biomedicines9010019)

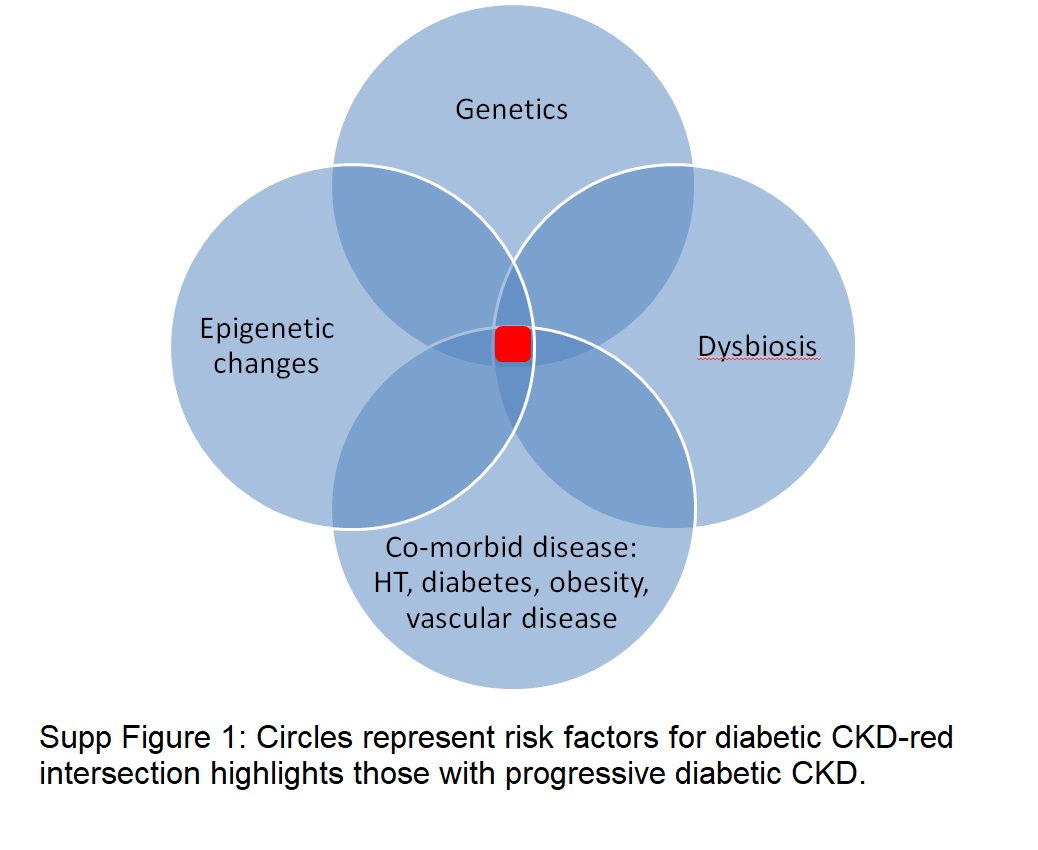

Supplement: Supplementary file 1 [file biomedicines-09-00019-s001.zip › Supp Figure 1.jpg]
